# Stable Composition of the Nano- and Picoplankton Community during the Ocean Iron Fertilization Experiment LOHAFEX

**DOI:** 10.1371/journal.pone.0113244

**Published:** 2014-11-17

**Authors:** Stefan Thiele, Christian Wolf, Isabelle Katharina Schulz, Philipp Assmy, Katja Metfies, Bernhard M. Fuchs

**Affiliations:** 1 Department of Molecular Ecology, Max Planck Institute for Marine Microbiology, Bremen, Germany; 2 Department of Polar Biological Oceanography, Division of Bioscience, Alfred Wegener Institute - Helmholtz Centre for Polar and Marine Research, Bremerhaven, Germany; 3 Bremen International Graduate School for Marine Sciences (GLOMAR), MARUM - Center for Marine Environmental Sciences, University of Bremen, Bremen, Germany; 4 Center for Ice, Climate and Ecosystems (ICE), Norwegian Polar Institute, Fram Centre, Tromsø, Norway; Instituto de Biologia, Brazil

## Abstract

The iron fertilization experiment LOHAFEX was conducted in a cold-core eddy in the Southern Atlantic Ocean during austral summer. Within a few days after fertilization, a phytoplankton bloom developed dominated by nano- and picoplankton groups. Unlike previously reported for other iron fertilization experiments, a diatom bloom was prevented by iron and silicate co-limitation. We used 18S rRNA gene tag pyrosequencing to investigate the diversity of these morphologically similar cell types within the nano- and picoplankton and microscopically enumerated dominant clades after catalyzed reported deposition fluorescence *in* *situ* hybridization (CARD-FISH) with specific oligonucleotide probes. In addition to *Phaeocystis*, members of Syndiniales group II, clade 10–11, and the *Micromonas* clades ABC and E made up a major fraction of the tag sequences of the nano- and picoplankton community within the fertilized patch. However, the same clades were also dominant before the bloom and outside the fertilized patch. Furthermore, only little changes in diversity could be observed over the course of the experiment. These results were corroborated by CARD-FISH analysis which confirmed the presence of a stable nano- and picoplankton community dominated by *Phaeocystis* and *Micromonas* during the entire course of the experiment. Interestingly, although Syndiniales dominated the tag sequences, they could hardly be detected by CARD-FISH, possibly due to the intracellular parasitic life style of this clade. The remarkable stability of the nano- and picoplankton community points to a tight coupling of the different trophic levels within the microbial food web during LOHAFEX.

## Introduction

Phytoplankton blooms occur seasonally in large parts of the oceans. Typically, a spring or upwelling bloom dominated by large diatoms is followed closely by a community dominated by small nanoplankton. However, wide ocean areas exhibit low phytoplankton standing stocks despite perennially high nutrient concentrations. Such high nutrient - low chlorophyll areas (HNLCs) are present in the subarctic and equatorial Pacific Ocean but also in most of the Southern Ocean. John Martin and colleagues postulated in the early 1990-ies that iron availability limits phytoplankton growth in these HNLC areas [1]. In the following years a dozen Lagrangian experiments in iron-limited HNLC waters have shown that phytoplankton blooms can be induced by artificial iron fertilization [2]. In most experiments the iron induced blooms were dominated by large diatoms which stimulated the idea that the export of rapidly sinking diatom aggregates from iron-induced blooms could enhance the strength and efficiency of the biological carbon pump [3]. Additionally, the enhanced primary production in surface waters would lead to an increase of dissolved organic carbon (DOC) and particulate organic carbon (POC), both of which are the basis nutrition for different levels of the microbial loop, in particular the bacterial and archaeal community [4]. To quantify the extent of carbon export of phytoplankton biomass and the impact of the microbial loop in surface waters the Indo-German iron fertilization experiment LOHAFEX (‘loha’ is Hindi for ‘iron’; FEX for Fertilization EXperiment) was conducted in late austral summer of 2009 in a cold core eddy north of the Antarctic Polar Front in the Atlantic sector of the subantarctic Southern Ocean. In previous iron fertilization experiments the abundance and diversity of the large bloom-forming diatoms had been explored in detail [5]–[8], while smaller Eukarya, ranging from 2–20 µm (nanoplankton) and 0.2–2 µm (picoplankton), have been rarely explored and were treated as “black boxes” in most of the studies so far. Eukaryotic nano- and picoplankton have been observed to dominate blooms after iron-fertilization [9], [10], especially in areas with a co-limitation of iron and silicate. For example during the SAGE iron fertilization experiment, haptophytes and prasinophytes accounted for ∼75% of the chlorophyll *a* content [11]. During LOHAFEX mainly *Phaeocystis*-like small flagellated and non-flagellated taxa dominated the bloom upon fertilization and only little export could be measured which was possibly the consequence of the co-limitation of dissolved iron and silica in the fertilized patch [12]–[14].

With this study we identified and quantified the response of the eukaryotic nano- and picoplankton during the LOHAFEX experiment. Several methods are available for identification and quantification. The most wide-spread method is to count cells in Lugol- or formaldehyde-fixed water samples settled in sedimentation chambers by inverted light microscopy and to quantify total cell numbers based on different size classes and morphologies [15]. The cell numbers of eukaryotic nano- and picoplankton during LOHAFEX have been quantified by this method and are reported in detail in an accompanying study [12]. However with the exception of a few morphologically distinct species, quantification of specific groups of these otherwise featureless small eukaryotes remains problematic. Scanning electron microscopy provides more morphological details due to higher resolution [16], yet it is not suited for high throughput analyses.

Molecular biological tools based on ribosomal RNA genes, like catalysed reporter deposition (CARD-) fluorescence *in* *situ* hybridization (FISH) [17], [18] and tag pyrosequencing [19], provide a stable phylogenetic framework with a resolution superior to that of other molecular methods such as marker pigment analyses [20]. Using the rRNA approach, a wealth of previously unexplored diversity was recently revealed from different ocean areas [21]–[24]. FISH is well established for the identification and quantification of Bacteria and Archaea in complex environmental samples, and was also successfully applied to investigate eukaryotic nano- and picoplankton communities [22], [25]. A combined approach using sequencing and FISH methods is commonly used for the identification of bacterial and archaeal communities [17]. Therefore in this study we aimed at combining methods established for nano- and picoplankton analyses, like light microscopic quantification of Lugol-fixed samples with tag pyrosequencing [19] and FISH [18] to characterize the eukaryotic nano- and picoplankton community composition with higher taxonomic resolution. This combination of methods has also the capacity to tap into yet unknown diversity and to discover novel organisms involved in iron-induced phytoplankton blooms.

## Material & Methods

### Sampling

**Figure 1:**
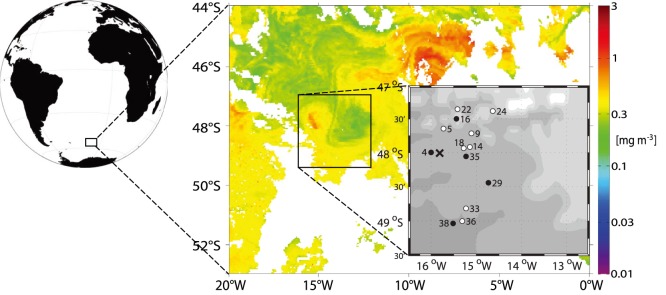
Map and sampling scheme of LOHAFEX. MODIS (Moderate Resolution Imaging Spectroradiometer) satellite image from 14. February 2009 showing chlorophyll *a* concentrations for the Polar Frontal Zone with the LOHAFEX bloom encircled. Stations and experiment days of both the IN (black) and OUT stations (white) are shown in the small map. The X marks day −1 before the iron addition on 27^th^ January. The globe and the inset map were generated with the M_Map package for Matlab (version 7.12.0.635; MathWorks, Natick, MA). The chlorophyll *a* data were downloaded from the NASA website http://oceancolor.gsfc.nasa.gov/.

The iron fertilization experiment LOHAFEX was conducted during the RV “Polarstern” cruise ANT XXV/3 (12^th^ January to 6^th^ March, 2009) as described previously [13], [26]. Briefly, the closed core of a stable cyclonic eddy adjacent to the Antarctic Polar Front in the Atlantic sector of the subantarctic Southern Ocean was fertilized with 2 t of Fe (10 t of FeSO_4_×7 H_2_O) on 27^th^ January. A second fertilization was applied using 2 t of Fe (10 t of FeSO_4_×7 H_2_O) after 18 days (on 14^th^ February). The fertilized patch was monitored for 38 days. As a response to the fertilization, Fv/Fm ratios increased from below 0.3 to above 0.45 and chlorophyll *a* concentrations increased from 0.5 µg l^−1^ to 1.0–1.2 µg l^−1^ within 14 days [12], [13]. The peak chlorophyll value of 1.6 µg l^−1^ was reached at the end of the third week. Both Fv/Fm and chlorophyll values decreased thereafter to values of 0.35 and 0.7 µg l^−1^, respectively. Samples were taken on day −1 prior to the start of the experiment, on days 5, 9, 14, 18, 22, 24, 33, 36 inside the fertilized patch (“IN” stations) and days 4, 16, 29, 35 (only 20 m), and 38 outside the fertilized patch (“OUT” stations, Figure 1) [12]. Both IN and OUT stations were situated within the eddy. On each day, 190 ml of water from 20 m depth and 40 m depth were fixed with 10 ml acidic Lugol solution (5% final conc. v/v) and stored in brown glass bottles at 4°C in the dark for 1.5–2.5 years until manual counting and 3 years until CARD-FISH analysis. Due to the well mixed water column from surface down to 60–80 m depth (data not shown), both samples (20 and 40 m) were treated as replicates. For DNA extraction 90 l (day −1), 85 l (day 9), 75 l (day 16/OUT), and 67 l (day 18) were sampled at 20 m depth and filtered on 0.2 µm pore size cellulose acetate filters (Sartorius, Göttingen, Germany) after a prefiltration step with a 5 µm. filter. These samples were stored at −80°C.

### DNA extraction and tag-pyrosequencing

DNA extraction was done using the E.Z.N.A. SP Plant DNA Kit (Omega Bio-Tek, Norcross, USA). Initially, the filters were incubated in lysis buffer (provided in the kit) at 65°C for 10 min before performing all further steps as described in the manufacturer’s instructions. The eluted DNA was stored at −20°C until further analysis. We amplified ∼670 bp fragments of the 18S rRNA gene, containing the highly variable V4-region, using the primer-set 528F (5′-GCGGTAATTCCAGCTCCAA-3′) and 1055R (5′-ACGGCCATGCACCACCACCCAT-3′) modified after Elwood [27] as described by Wolf [28]. Pyrosequencing (single reads, forward direction) was performed on a Genome Sequencer FLX system (Roche, Penzberg, Germany) by GATC Biotech AG (Konstanz, Germany). Raw sequence reads were processed to obtain high quality reads (Table S1). Reads with a length below 300 bp, reads longer than >670 bp and reads with more than one uncertain base (N) were excluded from further analysis. Chimera sequences were excluded using the software UCHIME 4.2 [29]. The high quality reads of all samples were clustered into operational taxonomic units (OTUs) at the 98% identity level using the SILVAngs pipeline (https://www.arb-silva.de/ngs/; [30]) (Table S1). Consensus sequences of each OTU were generated and used for further analyses. The 98% identity level is conservative, but was found suitable to reproduce original eukaryotic diversity [31] and to embrace the error-rate of 454 pyrosequencing. The consensus sequences were aligned and imported into a manually curated reference tree containing 51.553 high quality sequences of Eukarya of the SILVA reference database (release SSU_Ref_119, July 2014) by parsimony criteria using the ARB software suite [30]. Classification was done based on the resulting positioning of the consensus sequence in the tree. The raw sequence data generated in this study has been deposited at GenBank’s Short Read Archive (SRA) under the accession number SRA064723.

### CARD-FISH

In tests, the margins of many Lugol-fixed cells appeared disrupted, shrunken or shapeless after CARD-FISH and indicated an elevated cell loss. Therefore, an additional fixation step with formaldehyde was introduced to further stabilize the cells for CARD-FISH and to ensure bright signals and stable cell counts. Hundred milliliter of Lugol-fixed sample was incubated for 1 h with formaldehyde (1% final concentration), neutralized with 1 M sodium thiosulfate and filtered onto polycarbonate filters with 0.8 µm pore size after pre-filtration using 20 µm pore size filters (Millipore, Tullagreen, Ireland). Due to limited sample amount, only 25 ml and 70 ml were filtered for samples from day −1 and day 38 (both 20 m depth).

CARD-FISH was done as described previously [32]. Briefly, samples were embedded in 0.1% agarose. A permeabilization step was done with Proteinase K (5 µg/ml) for 15 minutes for hybridizations with the probe PHAEO03 due to the length of 34 bp of this probe. Hybridization and amplification was done on glass slides using 50 ml tubes or in Petri dishes using 700 ml glass chambers as moisture chambers at 46°C. We used 14 horseradish peroxidase (HRP) labeled oligonucleotide probes (Table 1) including the probe NON338 as a negative control. All other probes were chosen according to 454 tag sequencing results. For signal amplification, Alexa488 labeled tyramides were used for all probes. After the CARD procedure samples were stained with DAPI for quantification of total cell numbers.

**Table 1:** List of oligonucleotides used in this study.

Probe	Target organism	Sequence (5′→3′)	FA (%)^a^	Reference
**EUK516**	Eukarya	ACCAGACTTGCCCTCC	0	[17]
**NON338**	Control	ACTCCTACGGGAGGCAGC	35	[45]
**PRAS04**	Mamiellophyceae	CGTAAGCCCGCTTTGAAC	40	[38]
**PRYM02**	Haptophyta	GGAATACGAGTGCCCCTGAC	40	[46]
**MICRO01**	*Micromonas pusilla*	AATGGAACACCGCCGGCG	40	[38]
**PHAEO03**	*Phaeocystis*	GAGTAGCCGCGGTCTCCGG AAAGAAGGCCGCGCC	20	[47]
**PELA01**	Pelagophyceae	GCAACAATCAATCCCAATC	20	[46]
**MAST1A**	MAST 1 clade	ATTACCTCGATCCGCAAA	30	[22]
**MAST1B**	MAST 1 clade	AACGCAAGTCTCCCCGCG	30	[22]
**MAST1C**	MAST 1 clade	GTGTTCCCTAACCCCGAC	30	[22]
**MAST3**	MAST 3	ATTACCTTGGCCTCCAAC	30	[48]
**MAST4**	MAST 4	TACTTCGGTCTGCAAACC	30	[48]
**SYN-I-1161**	Syndiniales group I	TCCTCGCGTTAGACACGC	20	This study
**SYN-II-675**	Syndiniales group II	CACCTCTGACGCGTTAAT	20	This study

A Probe-check of PRAS04 on SILVA ref 119 targeted 95% for the class Mamiellophyceae [38] with only one false-positive hit in the Dinophyceae and one in the Chrysophyceae, but no other hits in the Prasinophyceae. Thus, probe PRAS04 is specific only for Mamiellophyceae (Figure S3). Similarly, probe SYN-I-1161 resulted in a 30% coverage of the Syndiniales group I (48 outgroup hits) and SYN-II-675 targeted 42% of Syndiniales group II (no outgroup hits). However, SYN-II-675 targeted 82% of the Syndiniales group II clade 10–11, the main Syndiniales clade during LOHAFEX.

a Formamide concentration in the CARD-FISH hybridisation buffer.

### Cell quantification

For nano- and picoplankton cell quantification, two different methods were used. Quantification of CARD-FISH positive cells was done manually on an Eclipse 50i microscope (Nikon, Amstelveen, Netherland) at 1000x magnification in 50 fields of view (FOV) per sample in duplicates (Table S2). Total cell numbers were counted from the same CARD-FISH preparations using an automated counting routine. A Zeiss AxioImager. Z2 microscope (Zeiss, Jena, Germany) equipped with an automated stage was used to automatically acquire images from the preparations using the software package AxioVision Release 4.7 (Zeiss, Jena, Germany) and the macro [33] MPISYS. Image acquisition comprised an automated focusing routine, an automated sample area definition and a manual image quality assessment [33]. The software takes three images of each field of view along a given track on the sample, one in the DAPI channel (350 nm), one in the FISH channel (488 nm), and one at 594 nm at the main autofluorescence of the cells, caused by various cell components which were not further analyzed (Figure S1). These picture triplets were further processed using the software ACMEtool 0.76 (an updated version including description is available on www.technobiology.ch). Before processing further, a manual quality check was done for every picture triplet and non-usable triplets were discarded. After this quality control, cells were detected automatically using an algorithm optimized for nano- and picoplankton quantification in the ACMEtool 0.76. Since DAPI signals were often quenched by strong autofluorescence and not all cells were stained by the general eukaryotic probe EUK516, the algorithm combines the green probe signal, the orange autofluorescence of accessory pigments and the green autofluorescence of the cells to define nano- and picoplankton cells (Figure S1). After automatic cell detection, the pictures were again manually evaluated to include cells missed by the evaluation algorithm and then all cells were quantified. For quantification only samples with a minimum of 15 image triplets were considered. Total nano- and picoplankton cell counts were calculated as a mean value from a minimum of 13 CARD-FISH preparations.

### Probe design

Two new probes for the subclades I and II of the Syndiniales clade (Table 1) were designed using the probe design function of ARB [30] based on the SILVA ref 108 database [30] including the consensus sequences from tag pyrosequencing. Re-evaluations of the probes were done based on the SILVA ref 119 database from July 2014. Probe SYN-I-1161 had 48 target hits outside the Syndiniales group I (28 in dinoflagelates and 2 in Syndiniales group II, the rest scattered through the Eukarya). Probe SYN-II-675 showed no false-positives outside the Syndiniales group II. Optimal stringency of the probes was tested *in* *situ* by a series of increasing formamide concentration in the CARD-FISH buffer on a sample from day 38 (20 m depth) (Table 1).

### Statistics

The total cell numbers obtained by manual counting using light microscopy and automated counting were compared using linear regressions. Normal distribution of the data was tested using the Kolmogorov-Smirnov test. Normal distributed data were tested using one way ANOVAs including Holm-Sidak comparison and not-normal distributed data were tested using ANOVA on ranks. All analyses were done using SigmaStat 3.5 (Statcon, Witzenhausen, Germany).

## Results

### Community composition

**Figure 2:**
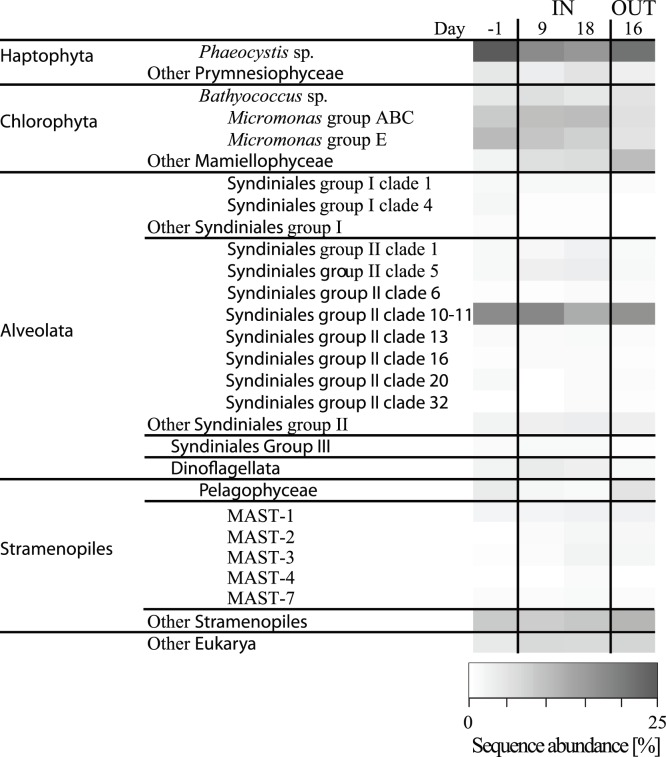
18S rRNA tag frequency for the most abundant OTUs. An abundant OTU contains >100 sequences at least at one sampling point. Less abundant OTUs were summarized into ‘Other [taxon]’.

The diversity of eukaryotic nano- and picoplankton was assessed by tag pyrosequencing in the 0.2–5 µm fraction one day before the start of the experiment, during the experiment on days 9 and 18 inside the fertilized patch, and on day 16 outside the fertilized patch (Figure 2). All four samples had a similar composition with respect to the abundant OTUs. The most frequent tags in all samples originated from Alveolata (31–37%), Chlorophyta (24–29%), Haptophyta (19–27%), and Stramenopiles (14–21%). Some of the 22 abundant OTUs (>100 reads/OTU) showed fluctuations in sequence abundance over the course of the experiment, while in general the community was rather stable (Figure 2). The frequency of sequence tags originating from members of the genus *Phaeocystis* (Haptophyta) decreased from 23% (day −1) to 15% (day 18, Figure 2). Among the Mamiellophyceae, a class within the Chlorophyta, the genus *Micromonas* was quite frequent within the induced bloom (17–18%), but were considerably lower at day 16 (9%) outside the fertilized patch. Group E of *Micromonas* was slightly less abundant (4–10%) than *Micromonas* group ABC (5–10%) and both showed lowest abundance outside of the fertilized patch (Figure S2). Furthermore *Bathycoccus* sp. was found abundant (4–5%) in the Mamiellophyceae. Pelagophyceae showed a decrease in sequence abundance from 3.3% at day −1 to 1.0% at day 18 inside the patch, while they were found in highest abundance at the OUT station on day 16, with 4.6% (Figure 2). The most important Alveolata were Syndiniales represented by group I, II, and III. Group I was dominated by clades 1 (∼1%) and clade 4 (0.2–1.7%), while the total sequence abundance of this group never exceeded 3.5% (Figure S3 A). The abundance of Syndiniales group III ranged around 1% with a minimum of 0.3% at day 16 outside the fertilized patch (Figure S3 A). Members of the Syndiniales group II were dominant inside and outside the bloom, showing relative abundance of 23–26%. The most dominant clade within this group was clade 10–11 with 12–17% relative abundance inside the fertilized patch (Figure S3 B). Clade 5 showed abundance of 1.2–3.1%, while clades 1, 6, 13, 16, 20, and 32 rarely occurred in abundance higher than 1.5% (Figure S3 B). Among the Stramenopiles, the most dominant OTU belonged to the MAST-1 clade (∼2%), and the MAST-3 clade (0.4–2.1%). The clades MAST-2, MAST-4 and MAST-7 were <1%. However, the bulk of OTUs within the Stramenopiles accounted for 4–10% of the sequences.

### Nano- and picoplankton cell numbers

**Figure 3:**
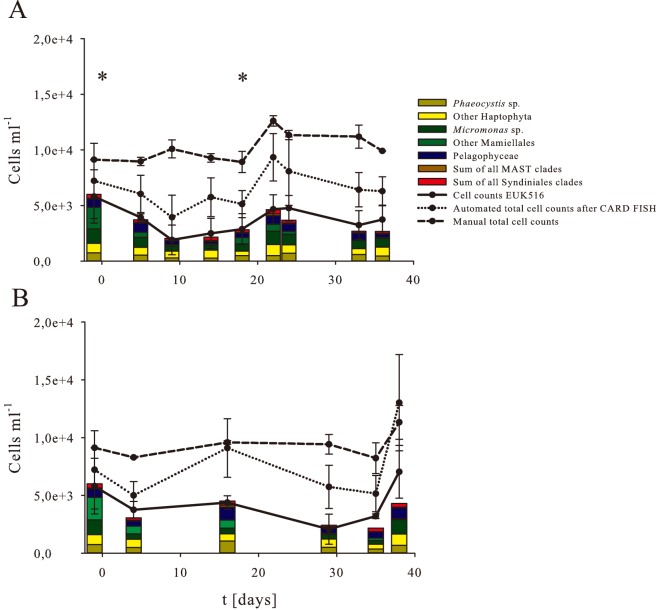
Quantification of the nano- and picoplankton community. Manual total cell counts from Lugol fixed samples (dashed lines), automated total cell counts after CARD-FISH (dotted lines), and cell counts of EUK516 probe (straight line) at IN (A) and OUT (B) stations. Stacked bar charts represent cell numbers of all other probes used in this study. Asterisks mark the iron fertilization events.

Nano- and picoplankton cells were first enumerated on Lugol fixed samples inside and outside the fertilized patch by manual counting using light microscopy [12]. In the mixed surface water layer nano- and picoplankton abundance was quite stable around 1.0×10^4^ ml^−1^±1.3×10^3^ cells ml^−1^ but increased slightly from 8.9×10^3^±3.7×10^2^ cells ml^−1^ on day 5 to 1.3×10^4^±4.6×10^2^ cells ml^−1^ on day 22 after the second iron addition. Cell numbers remained at this elevated level during the later phase of the experiment (Figure 3 A). Outside the fertilized patch cell numbers were almost identical to inside the patch in the early phase of the experiment and remained rather stable over the course of the experiment (9.4×10^3^±1.1×10^3^ cells ml^−1^). Only on the last day 38 of the experiment cell numbers increased to 1.1×10^4^±1.5×10^3^ cells ml^−1^ (Figure 3 B).

In comparison cell counts obtained with automated cell counting after CARD-FISH of Lugol- and formaldehyde fixed samples were by a factor of ∼1.5 (range 1.0–2.5) lower compared to the manual counts. We calculated that during the CARD-FISH procedure an average cell loss of 26%±11% occurred. However, similar to the manual counts, nano- and picoplankton abundance peaked on day 22 with 9.3×10^3^ cells ml^−1^, but otherwise cell numbers remained rather constant at 6.1±1.3×10^3^ cells ml^−1^ (Figure 3 A).

### Quantification of specific nano- and picoplankton clades

For the detection of nano- and picoplankton cells, the probe EUK516 was used in CARD-FISH, since it targets more than 85% of all Eukarya sequences in the SILVA ref NR 119 rRNA database. On average 60% of the nano- and picoplankton showed a positive signal after CARD-FISH with EUK516 in relation to the counts obtained by automated cell counting (Figure 3A+B). The numbers of EUK516 positive cells were highest on day −1 with 5.8×10^3^ cells ml^−1^, decreasing to 1.9×10^3^ cells ml^−1^ on day 9, before a second peak of 4.7×10^3^ cells ml^−1^and 4.8×10^3^ cells ml^−1^ on days 22 and 24 inside the fertilized patch (Figure 3 A). EUK-positive cell numbers were relatively constant outside the patch, but were as high as 7.0×10^3^ cells ml^−1^ on day 38, which was significantly different from the comparable IN station on day 36 (p = 0.045) (Figure 3 B).

To investigate the community structure of the nano- and picoplankton, we used CARD-FISH probes with nested specificity for different taxonomic clades based on the tag sequencing data. Within the nano- and picoplankton community inside the fertilized patch, Prymnesiophyceae, mainly from the genus *Phaeocystis* were the main contributors to the nano- and picoplankton community. However, abundance of *Phaeocystis* and other Prymnesiophyceae did not change significantly within the fertilized patch over the course of the experiment (Figure 3A). Values were constant at about 1.0×10^3^ cells ml^−1^ for all Prymnesiophyceae and 5.0×10^2^ cells ml^−1^ for *Phaeocystis* accounting for about 50% of the Prymnesiophyceae. At the OUT station on day 16, higher numbers of *Phaeocystis* were found with 1.1×10^3^ cells ml^−1^, consistent with significantly higher numbers of Prymnesiophyceae (1.5×10^3^ cells ml^−1^) (p = 0.01) (Figure 3B).

Mamiellophyceae, a second dominant class in the tag sequences, showed a higher variation in cell numbers inside the fertilized patch, ranging from 3.2×10^3^ cells ml^−1^ to 5.9×10^2^ cells ml^−1^, while cell numbers in the OUT stations remained rather constant and were significantly lower than inside the fertilized patch (p = 0.03). Cell numbers in the dominant subgroup *Micromonas* ranged from 1.3×10^3^ cells ml^−1^ to around 4.4×10^2^ cells ml^−1^. On average *Micromonas* accounted for ∼72% of the Mamiellophyceae (Figure 3A+B).

Pelagophyceae were also found rather stable in- and outside of the patch with numbers as high as 1.1×10^3^ cells ml^−1^ outside the patch on day 16 (Figure 3A+B). Abundance of the group Marine Stramenopiles (MAST) was low and never exceeded 1.7×10^2^ cells ml^−1^ during the course of the experiment (Figure 3A+B). Also the numbers of both Syndiniales clades were low and oscillated around 7.7×10^1^ cells ml^−1^ for Syndiniales clade I and around 1.8×10^2^ cells ml^−1^ for Syndiniales clade II within and outside of the fertilized patch, respectively (Figure 3A+B).

## Discussion

A striking outcome of the iron fertilization experiment LOHAFEX was that the phytoplankton standing stocks were dominated by the nano- and picoflagellates, while diatoms never contributed more than 5% [12], [13]. A similar response of the plankton community was found during the 15-day SAGE experiment, where diatoms were also co-limited by silicate and consequently picoplankton species dominated the planktonic community [11]. During LOHAFEX the cell numbers of nano- and picoplankton were remarkably stable and showed no large fluctuations during the experiment [12]. This was similar to the response of the bacterioplankton community reported earlier [26].

The diversity of the nano- and picoplankton community using 18S rRNA tag pyrosequencing did not change significantly in the samples analyzed. There were only minor differences between IN and OUT stations and the community composition was highly similar before and after the iron additions. The minor fluctuations found within the bloom could be also attributed to the inconsistencies inherent to every PCR-dependent assay. Consequently, CARD-FISH was used to check for any fluctuations in the major abundant groups of flagellates, which might have been missed by the tag sequencing analysis. We encounter several problems, which are summarized below.

When comparing the cell numbers gained by our automated microscopic cell counting routine with the cell numbers gained by direct light microscopic cell counting [12], we noticed consistently lower cell numbers of the former although both counts were done on the same samples obtained from the same CTD casts during the LOHAFEX experiment. The differences between the cell counts could be due to a number of reasons, maybe also a combination of them. During light microscopic counting, small coccoid cells tend to be underestimated, in contrast, biomass estimations could lead to overestimation of abundance due to cell shrinkage or swelling after fixation. For CARD-FISH, Lugol fixed samples had to be stabilised by additional fixation with formaldehyde [34]. The long storage time in Lugol solution for three years has likely led to cell loss of the more delicate cells. The subsequent filtration step could be an additional source of cell loss [35]. Some of the smaller picoplankton cells might have passed through the pores of 0.8 µm diameter of the polycarbonate filter [36] or cells might have been ruptured during filtration. Several washing steps during CARD-FISH might have led to cell loss, although the samples were embedded in a thin layer of agarose. However, the proportion of clades from tag sequencing and CARD-FISH were highly similar and therefore a preferential loss of a specific group of nano- or picoplankton cells is quite unlikely. However, future studies need to take preservation of these fragile cell types in consideration.

Existing probes were chosen for the dominant groups based on the tag sequencing data and, in the case of the Syndiniales, two new probes were designed. This was necessary to be able to distinguish between the two main Syndiniales groups I and II. Furthermore, Syndiniales probe SYN-II-675 was designed to match with clade 10–11, the most abundant clade during LOHAFEX. The relative abundance of CARD-FISH positive cells corroborated well the relative sequence representations from tag pyrosequencing. However, after CARD-FISH about 30–50% of the nano- and picoplankton cells showed no signal with the general probe EUK516, which was used as a positive control. Most likely this was due to quenching of the probe-conferred fluorescence by elevated autofluorescence of the cells. Alternatively, the accessibility of the ribosomes or even the number of ribosomes in the cells might have been reduced by Lugol fixation and thus no hybridization was possible. Nevertheless the sums for all clade-specific counts were in good agreement with the counts of the EUK516 probe (∼90%) and demonstrate that we did not miss a major group of nano- or picoplankton.

Surprisingly during LOHAFEX Pelagophyceae and several MAST clades were found in relatively low abundance, even though ribosomal RNA studies show that members of the class Pelagophyceae (Stramenopiles) were reported frequently as major components of marine nano- and picoplankton communities [22]. Instead, the three most prominent bloom forming clades during the LOHAFEX experiment belonged to the Prymnesiophyceae, Mamiellophyceae, and Alveolata. Both, Prymnesiophyceae and Mamiellophyceae, made up 46–51% of the nano- and picoplankton community. Gomez-Pereira and coworkers found similar numbers of Mamiellophyceae in the same region [37]. The only *Micromonas* species, *M. pusilla*, was often dominating in phytoplankton blooms in the British Channel [38], in Pacific coastal waters and in Arctic waters [23], [39], but to our knowledge have been detected only in low numbers in the Southern Ocean so far [28]. During LOHAFEX this species, together with *Phaeocystis*, dominated the iron-induced phytoplankton bloom.

*Phaeocystis*, a genus of the Prymnesiophyceae, forms large blooms worldwide [40]. During LOHAFEX the bulk of *Phaeocystis* biomass was allocated to solitary cells but formation of colonies attached to diatoms and small free-floating colonies were also observed [12]. The discrepancy between light microscopic (∼10^4^ ml^−1^) and CARD-FISH counts (∼10^3^ ml^−1^) shows the difficulties in counting solely based on morphological features and underpins the necessity to further characterize the clade *Phaeocystis* by molecular tools.

Within the Alveolata, three Syndiniales groups were among the dominant organisms by tag pyrosequencing. Syndiniales were found in the Ross Sea before, though in lower abundance [27]. Most abundant among all OTUs was the Syndiniales group II clade 10–11, while other clades and groups were found only in minor abundance. With the newly developed CARD-FISH probes we could detect Syndiniales only in relatively low abundance, both, inside and outside the fertilized patch. A possible explanation might be that members of the Syndiniales group have been described as endosymbionts and parasites within algae, tintinnids, crustaceans and other Dinophyceae [41], although free-living cells can occur in abundance [42], [43]. Cells residing inside these organisms might be inaccessible for large HRP-labeled oligonucleotide probes, although the probe SYN-II-675 targets 82% of the sequences of the dominant Syndiniales group II clade 10–11 in the SILVA database. Syndiniales might have multiple 18S rRNA gene copies per cell, similar to the closely-related group of Dinoflagellata [44], which would partly explain the observed overrepresentation of Syndiniales in the tag sequences.

The taxonomically resolved monitoring of important components of the microbial loop during the LOHAFEX experiment revealed a surprising compositional stability. This stability is most likely caused by silicate limitation of diatoms and the absence of salps in the fertilized waters. While experimentally determined growth rates of diatoms were rather high, the low silicate concentration (<2 µM) were setting a low upper for diatom biomass build-up inside the fertilized patch [2]. It might be speculated, that salp grazing might have exerted a top down control on the nano- and picoplankton community. However, the salp abundance was low, most likely due to predation by *Themisto gaudichaudii* (Smetacek pers. communication). Due to these factors, nano- and picoplankton species were able to maintain high numbers and control the bacterial community [26], though no significant increase or change in diversity was found for the nano- and picoplankton community. It can be speculated that the lack of a pronounced increase by nano- and picoplankton inside the fertilized patch was due to top down control by dinoflagellates, naked ciliates, and tintinnids which were themselves kept in check by high numbers of copepods. During LOHAFEX, the whole planktonic ecosystem, and in particular the microbial loop, seemed to be tightly coupled, resulting in a strong cycling of carbon compounds within the microbial loop, that hence counteracted the efficiency of the biological carbon pump [11], [13].

## Supporting Information

Figure S1 **Picture triplets obtained using the macro MPISYS.** Three pictures from the same field of view taken in different channels with excitation light of different wavelength (DAPI: 365 nm, CARD-FISH: 470 nm and autofluorescence: 590 nm), using the probes PRAS04 (Mamiellophyceae) and PHAEO03 (*Phaeocystis*).
(PDF)

Figure S2 **18S rRNA-based tree reconstructions of the Syndiniales groups.** Tree in Figure S2A shows the different groups within the Syndiniales with a special focus on clades within the group I, while tree in Figure S2B displays clades of the Syndiniales group II. The trees were built using the ARB SILVA ref 119 database [30], calculated using Maximum Likelihood and Neighbour Joining algorithm. The aligned consensus tag sequences were added with parsimony criteria to the trees and percentage of tags falling into the respective clade are given behind the clades. Values in the wedges represent the number of reference sequences. Scale bar represents 5% and 1% estimated base substitution.
(PDF)

Figure S3 **18S rRNA-based tree reconstruction of the Mamiellales clades.** Values in the wedges represent the number of reference sequences, while values behind the clades show the abundance of LOHAFEX sequences in these clades The tree was build using the ARB SILVA ref 119 database [30], calculated using Neighbour Joining and Maximum Likelihood algorithms. The aligned consensus tag sequences were added with parsimony criteria to the trees and percentage of tags falling into the respective clade are given behind the clades. Values in the wedges represent the number of reference sequences. Scale bar represents 1% estimated base substitution.
(PDF)

Table S1 **Summary statistics of pyrosequencing reads.** The table also contains values after quality filtering and number of OTUs of the 0.2–5 µm size fraction.
(PDF)

Table S2 **Total cell numbers.** Results of the quantification of all probes at 20 m (2A) and 40 m (2B) depth. Counts for SYN-I-1161 and SYN-II-675 were not determined at day 29 OUT at 20 m and SYN-II-675 were not determined for day 29 OUT at 40 m (n.d.).
(PDF)

## References

[1] Martin JH (1990) Glacial-interglacial CO_2_ change: The iron hypothesis. Paleoceanography 5: 1–13.

[2] Boyd PW, Jickells T, Law CS, Blain S, Boyle EA, et al (2007) Mesoscale Iron Enrichment Experiments 1993–2005: Synthesis and Future Directions. Science 315: 612–617. 17272712 10.1126/science.1131669

[3] Ducklow HW, Steinberg DK, Buessler KO (2001) Upper ocean carbon export and the biological pump. Oceanography 14: 50–58.

[4] Azam F, Fenchel T, Field J, Gray J, Meyer L, et al (1983) The ecological role of water column microbes in the sea. Mar Ecol Prog Ser 10: 257–263.

[5] Gall MP, Boyd PW, Hall J, Safi KA, Chang H (2001) Phytoplankton processes. Part 1: Community structure during the Southern Ocean Iron RElease Experiment (SOIREE). Deep-Sea Res Pt II 48: 2551–2570.

[6] Tsuda A, Kiyosawa H, Kuwata A, Mochizuki M, Shiga N, et al (2005) Responses of diatoms to iron-enrichment (SEEDS) in the western subarctic Pacific, temporal and spatial comparisons. Prog Oceanograph 64: 189–205.

[7] Assmy P, Henjes J, Klaas C, Smetacek V (2007) Mechanisms determining species dominance in a phytoplankton bloom induced by the iron fertilization experiment EisenEx in the Southern Ocean. Deep-Sea Res Pt I 54: 340–362.

[8] Smetacek V, Klaas C, Strass VH, Assmy P, Montresor M, et al (2012) Deep carbon export from a Southern Ocean iron-fertilized diatom bloom. Nature 487: 313–319. 22810695 10.1038/nature11229

[9] Hall JA, Safi K (2001) The impact of in situ Fe fertilisation on the microbial food web in the Southern Ocean. Deep-Sea Res Pt II 48: 2591–2613.

[10] Coale KH, Johnson KS, Chavez FP, Buesseler KO, Barber RT, et al (2004) Southern Ocean iron enrichment experiment: Carbon cycling in high- and low-Si waters. Science 304: 408–414. 15087542 10.1126/science.1089778

[11] Peloquin J, Hall J, Safi K, Smith WO Jr, Wright S, et al (2011) The response of phytoplankton to iron enrichment in Sub-Antarctic HNLCLSi waters: Results from the SAGE experiment. Deep-Sea Res Pt II 58: 808–823.

[12] Schulz IK (2013) Mechanisms determining species succession and dominance during an iron-induced phytoplankton bloom in the Southern Ocean (LOHAFEX). Doctoral thesis, University Bremen, Germany. Available: http://nbn-resolving.de/urn:nbn:de:gbv:46-00103521-10. Accessed 15^th^ October 2014.

[13] Martin P, van der Loeff MR, Cassar N, Vandromme P, d’ Ovidio F, et al (2013) Iron fertilization enhanced net community production but not downward particle flux during the Southern Ocean iron fertilization experiment LOHAFEX. Global Biogeochem Cy 27: 871–881.

[14] Ebersbach F, Assmy P, Martin P, Schulz I, Wolzenburg S, et al (2014) Particle flux characterisation and sedimentation patterns of protistan plankton during the iron fertilisation experiment LOHAFEX in the Southern Ocean. Deep Sea Research Part I: Oceanographic Research Papers 89: 94–103.

[15] Utermöhl H (1958) Zur Vervollkommnung der quantitativen Phytoplankton-Methodik. Mitt int Ver theor angew Limnol 9: 1–38.

[16] Vørs N, Buck KR, Chavez FP, Eikrem W, Hansen LE, et al (1995) Nanoplankton of the equatorial Pacific with emphasis on the heterotrophic protists. Deep-Sea Res Pt II 42: 585–602.

[17] Amann RI, Ludwig W, Schleifer KH (1995) Phylogenetic identification and in situ detection of individual microbial cells without cultivation. Microbiol Rev 59: 143–169. 7535888 10.1128/mr.59.1.143-169.1995 PMC239358

[18] Pernthaler A, Pernthaler J, Amann R (2002) Fluorescence in situ hybridization and catalyzed reporter deposition for the identification of marine bacteria. Appl Environ Microbiol 68: 3094–3101. 12039771 10.1128/AEM.68.6.3094-3101.2002 PMC123953

[19] Ronaghi M, Karamohamed S, Pettersson B, Uhlén M, Nyrén P (1996) Real-Time DNA sequencing using detection of pyrophosphate release. Analyt Biochem 242: 84–89. 8923969 10.1006/abio.1996.0432

[20] Mackey M, Mackey D, Higgins H, Wright S (1996) CHEMTAX - a program for estimating class abundances from chemical markers: application to HPLC measurements of phytoplankton. Mar Ecol Prog Ser 144: 265–283.

[21] Not F, Simon N, Biegala IC, Vaulot D (2002) Application of fluorescent in situ hybridization coupled with tyramide signal amplification (FISH-TSA) to assess eukaryotic picoplankton composition. Aquat Microb Ecol 28: 157–166.

[22] Massana R, Terrado R, Forn I, Lovejoy C, Pedrós-Alió C (2006) Distribution and abundance of uncultured heterotrophic flagellates in the world oceans. Environ Microbiol 8: 1515–1522. 16913912 10.1111/j.1462-2920.2006.01042.x

[23] Kilias E, Wolf C, Nöthig EM, Peeken I, Metfies K (2013) Protist distribution in the Western Fram Strait in summer 2010 based on 454-pyrosequencing of 18S rDNA. J Phycol 49: 996–1010. 27007321 10.1111/jpy.12109

[24] Unrein F, Gasol JM, Not F, Forn I, Massana R (2014) Mixotrophic haptophytes are key bacterial grazers in oligotrophic coastal waters. ISME J 8: 164–176. 23924785 10.1038/ismej.2013.132 PMC3869011

[25] Beardsley C, Knittel K, Amann R, Pernthaler J (2005) Quantification and distinction of aplastidic and plastidic marine nanoplankton by fluorescence in situ hybridization. Aquat Microb Ecol 41: 163–169.

[26] Thiele S, Fuchs BM, Ramaiah N, Amann R (2012) Microbial community response during the iron fertilization experiment LOHAFEX. Appl Environ Microbiol 78: 8803–8812. 23064339 10.1128/AEM.01814-12 PMC3502927

[27] Elwood HJ, Olsen GJ, Sogin ML (1985) The small-subunit ribosomal RNA gene sequences from the hypotrichous ciliates *Oxytricha nova* and *Stylonychia pustulata* . Mol Biol Evol 2: 399–410. 3939705 10.1093/oxfordjournals.molbev.a040362

[28] Wolf C, Frickenhaus S, Kilias ES, Peeken I, Metfies K (2013) Regional variability in eukaryotic protist communities in the Amundsen Sea. Antarctic Science 25: 741–751.

[29] Edgar RC, Haas BJ, Clemente JC, Quince C, Knight R (2011) UCHIME Improves Sensitivity and Speed of Chimera Detection. Bioinformatics 27: 2194–2200. 21700674 10.1093/bioinformatics/btr381 PMC3150044

[30] Quast C, Pruesse E, Yilmaz P, Gerken J, Schweer T, et al (2012) The SILVA ribosomal RNA gene database project: improved data processing and web-based tools. Nucl Acids Res 41: D590–D596. 23193283 10.1093/nar/gks1219 PMC3531112

[31] Behnke A, Engel M, Christen R, Nebel M, Klein RR, et al (2011) Depicting more accurate pictures of protistan community complexity using pyrosequencing of hypervariable SSU rRNA gene regions. Environ Microbiol 13: 340–349. 21281421 10.1111/j.1462-2920.2010.02332.x

[32] Thiele S, Fuchs B, Amann R (2011) Identification of microorganisms using the ribosomal RNA approach and fluorescence in situ hybridization. In: Wilderer P, editor. Treatise on Water Science. Oxford: Academic Press, Vol. 3: 171–189.

[33] Zeder M, Kohler E, Pernthaler J (2010) Automated quality assessment of autonomously acquired microscopic images of fluorescently stained bacteria. Cytometry A 77: 76–85. 19821518 10.1002/cyto.a.20810

[34] Sherr EB, Caron DA, Sherr BF (1993) Staining of heterotrophic protists for visualization via epifluorescence microscopy. In: Kemp PF, Cole JJ, Sherr BF, Sherr EB, editors. Handbook of Methods in Aquatic Microbial Ecology. Boca Raton, USA: CRC Press. 213–227.

[35] Bloem J, Bar-Gilissen MJB, Cappenberg TE (1986) Fixation, counting, and manipulation of heterotrophic Nanoflagellates. Appl Environ Microbiol 52: 1266–1272. 16347232 10.1128/aem.52.6.1266-1272.1986 PMC239220

[36] Gasol JM, Morn XAG (1999) Effects of filtration on bacterial activity and picoplankton community structure as assessed by flow cytometry. Aquat Microb Ecol 16: 251–264.

[37] Gómez-Pereira PR, Kennaway G, Fuchs BM, Tarran GA, Zubkov MV (2013) Flow cytometric identification of Mamiellales clade II in the Southern Atlantic Ocean. FEMS Microbiology Ecology 83: 664–671. 23035812 10.1111/1574-6941.12023

[38] Not F, Latasa M, Marie D, Cariou T, Vaulot D, et al (2004) A single species, *Micromonas pusilla* (Prasinophyceae), dominates the eukaryotic picoplankton in the western English Channel. Appl Environ Microbiol 70: 4064–4072. 15240284 10.1128/AEM.70.7.4064-4072.2004 PMC444783

[39] Balzano S, Marie D, Gourvil P, Vaulot D (2012) Composition of the summer photosynthetic pico and nanoplankton communities in the Beaufort Sea assessed by T-RFLP and sequences of the 18S rRNA gene from flow cytometry sorted samples. ISME J 6: 1480–1498. 22278671 10.1038/ismej.2011.213 PMC3400408

[40] Schoemann V, Becquevort S, Stefels J, Rousseau V, Lancelot C (2005) *Phaeocystis* blooms in the global ocean and their controlling mechanisms: a review. J Sea Res 53: 43–66.

[41] Coats DW, Adam EJ, Gallegos CL, Hedrick S (1996) Parasitism of photosynthetic dinoflagellates in a shallow subestuary of Chesapeake Bay, USA. Aquat Microb Ecol 11: 1–9.

[42] Chambouvet A, Morin P, Marie D, Guillou L (2008) Control of toxic marine dinoflagellate blooms by serial parasitic killers. Science 322: 1254–1257. 19023082 10.1126/science.1164387

[43] Siano R, Alves-de-Souza C, Foulon E, Bendif EM, Simon N, et al (2011) Distribution and host diversity of Amoebophryidae parasites across oligotrophic waters of the Mediterranean Sea. Biogeosciences 8: 267–278.

[44] Zhu F, Massana R, Not F, Marie D, Vaulot D (2006) Mapping of picoeucaryotes in marine ecosystems with quantitative PCR of the 18S rRNA gene. FEMS Microbiol Ecol 52: 79–92. 10.1016/j.femsec.2004.10.006 16329895

[45] Wallner G, Amann R, Beisker W (1993) Optimizing fluorescent in situ hybridization with rRNA-targeted oligonucleotide probes for flow cytometric identification of microorganisms. Cytometry 14: 136–143. 7679962 10.1002/cyto.990140205

[46] Simon N, Campbell L, Ornolfsdottir E, Groben R, Guillou L, et al (2000) Oligonucleotide probes for the identification of three algal groups by dot blot and fluorescent whole-cell hybridization. J Eukaryot Microbiol 47: 76–84. 10651300 10.1111/j.1550-7408.2000.tb00014.x

[47] Zingone A, Chretiennot-Dinet M, Lange M, Medlin L (1999) Morphological and genetic characterization of *Phaeocystis cordata* and *P. jahnii* (Prymnesiophyceae), two new species from the Mediterranean Sea. J Phycol: 1322–1337.

[48] Massana R, Guillou L, Díez B, Pedrós-Alió C (2002) Unveiling the Organisms behind Novel Eukaryotic Ribosomal DNA Sequences from the Ocean. Appl Environ Microbiol 68: 4554–4558. 12200313 10.1128/AEM.68.9.4554-4558.2002 PMC124113

