# Supplementary figures and images for: Stable Composition of the Nano- and Picoplankton Community during the Ocean Iron Fertilization Experiment LOHAFEX
Source: PLoS One. 2014 Nov 17;9(11):e113244. doi: 10.1371/journal.pone.0113244 (PMC4234645; doi:10.1371/journal.pone.0113244)

probe: PRAS04

probe: PHAEO03

DAPI

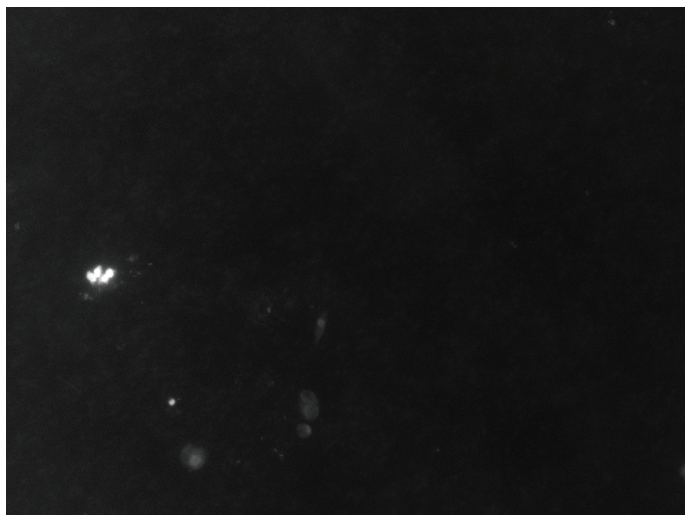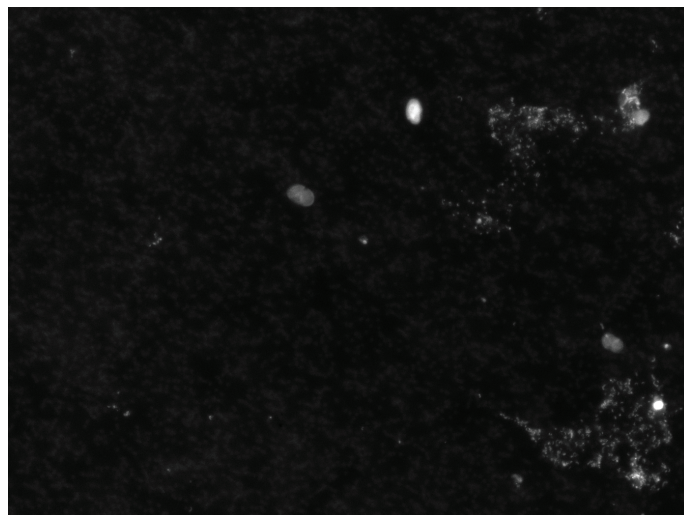

CARD FISH

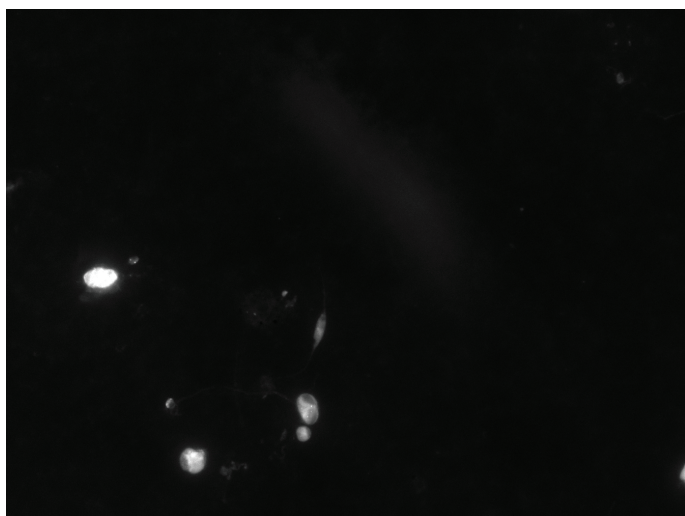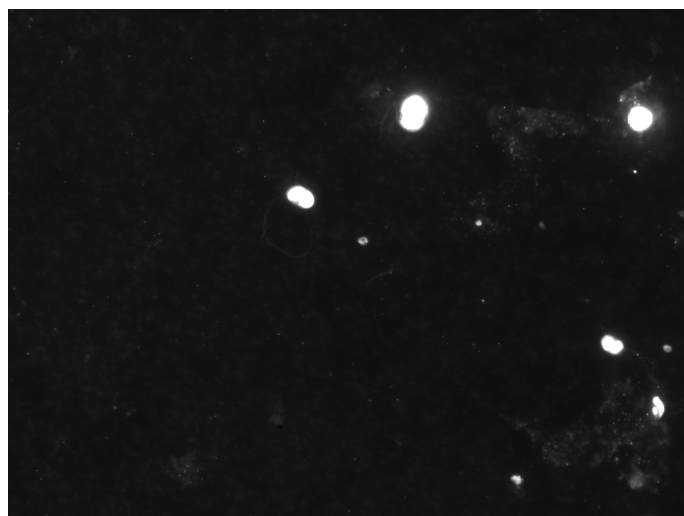

Autofluorescence

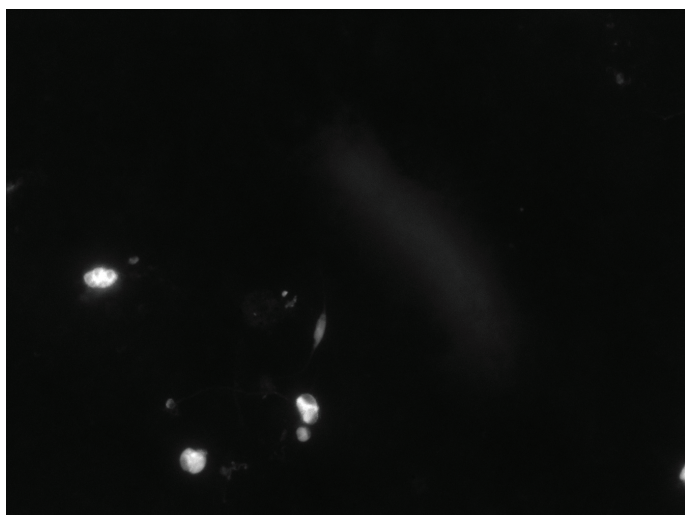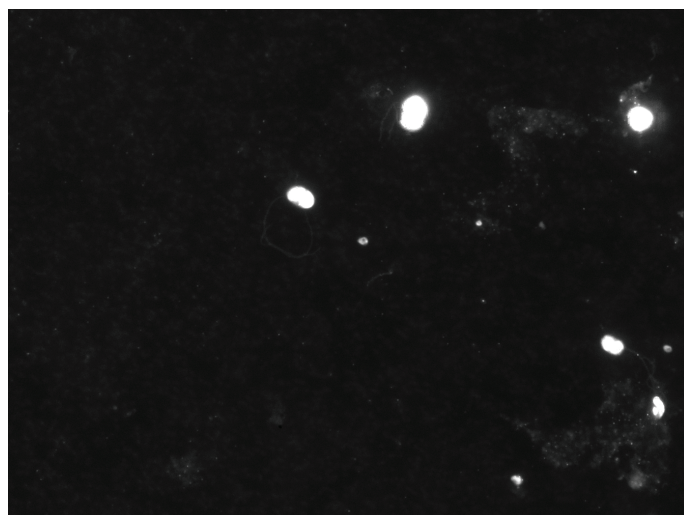

Supplement: Figure S1 — Picture triplets obtained using the macro MPISYS. Three pictures from the same field of view taken in different channels with excitation light of different wavelength (DAPI: 365 nm, CARD-FISH: 470 nm and autofluorescence: 590 nm), using the probes PRAS04 (Mamiellophyceae) and PHAEO03 (Phaeocystis). (PDF) [file pone.0113244.s001.pdf]

A

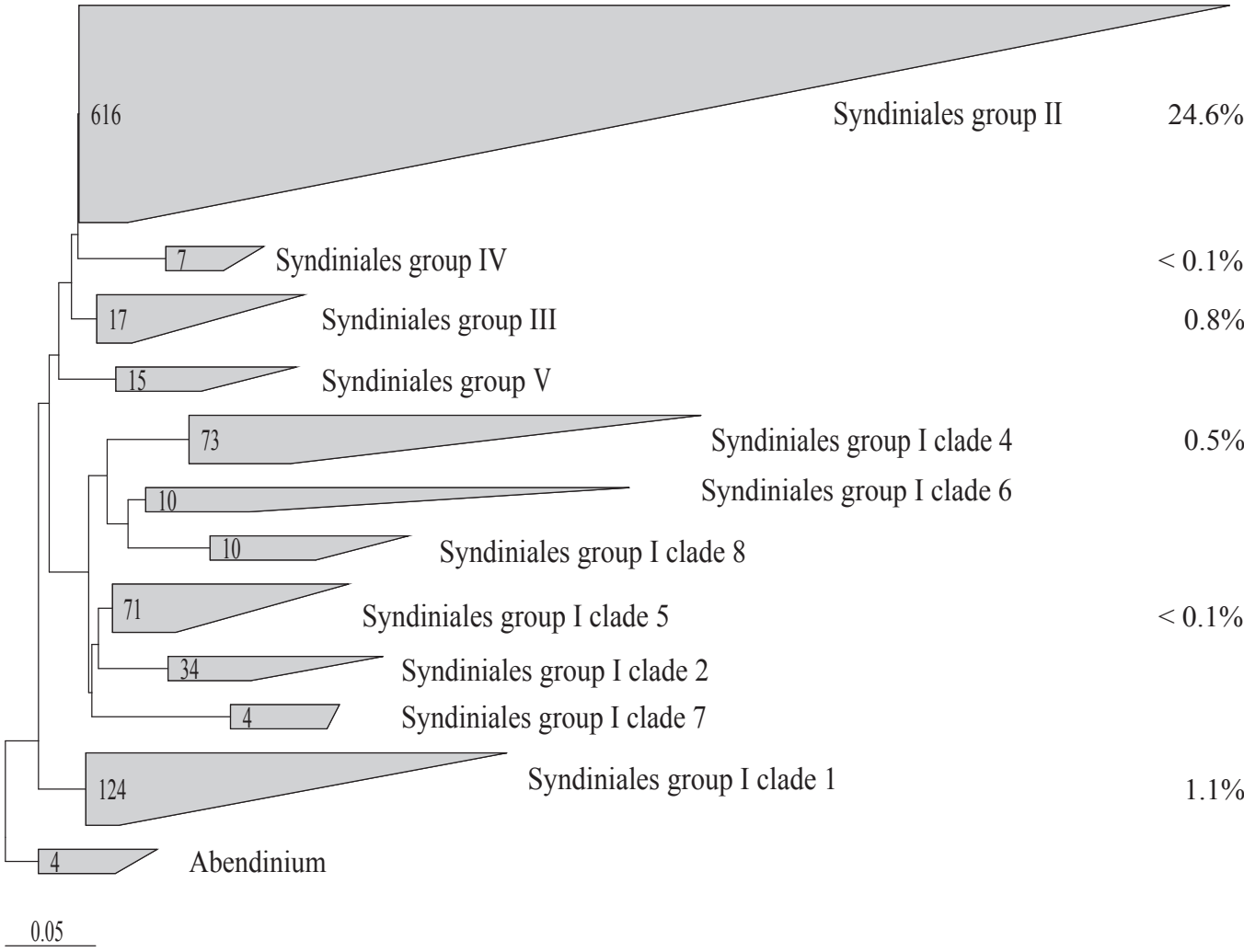

B

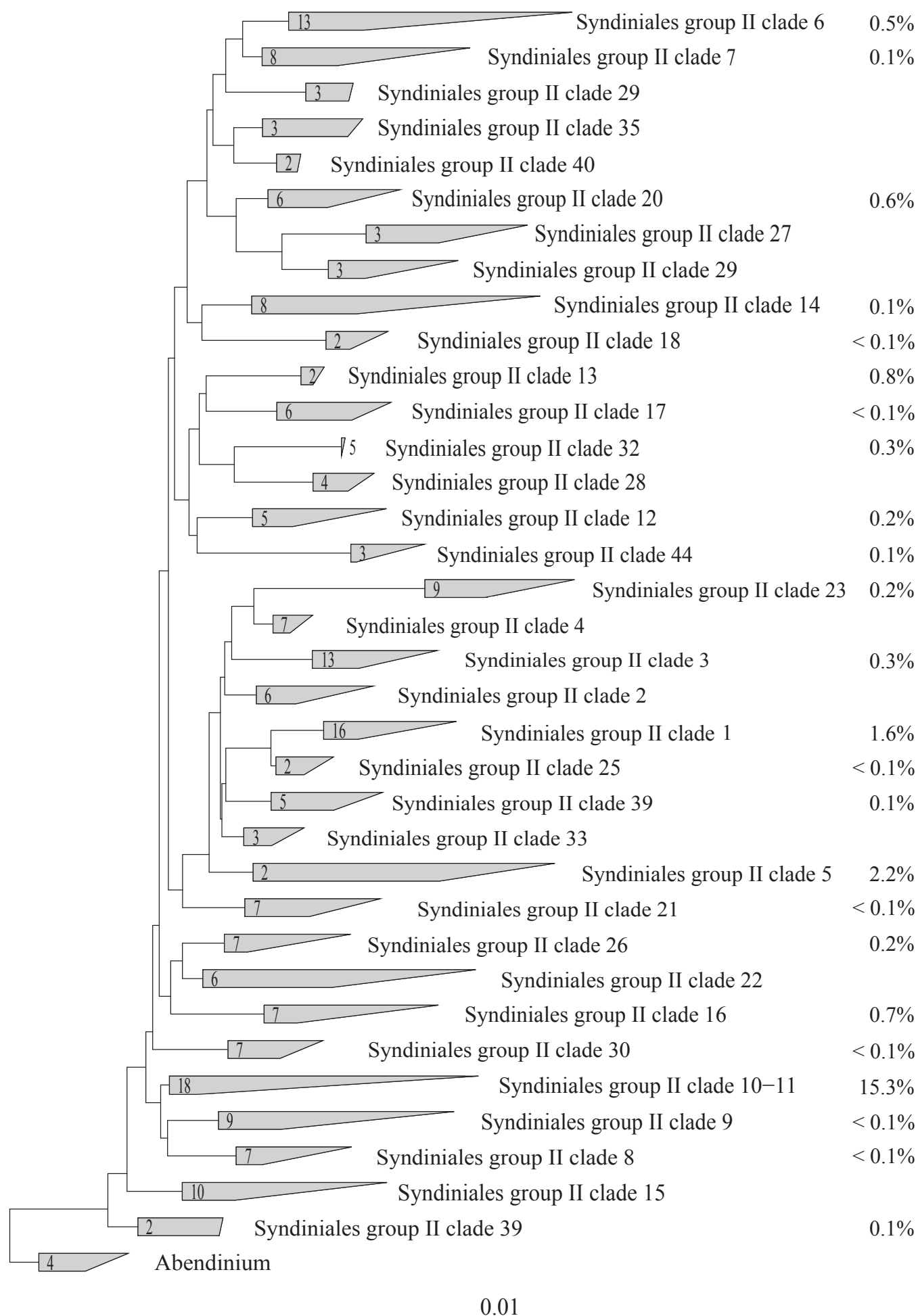

Supplement: Figure S2 — 18S rRNA-based tree reconstructions of the Syndiniales groups. Tree in Figure S2A shows the different groups within the Syndiniales with a special focus on clades within the group I, while tree in Figure S2B displays clades of the Syndiniales group II. The trees were built using the ARB SILVA ref 119 database [30], calculated using Maximum Likelihood and Neighbour Joining algorithm. The aligned consensus tag sequences were added with parsimony criteria to the trees and percentage of tags falling into the respective clade are given behind the clades. Values in the wedges represent the number of reference sequences. Scale bar represents 5% and 1% estimated base substitution. (PDF) [file pone.0113244.s002.pdf]

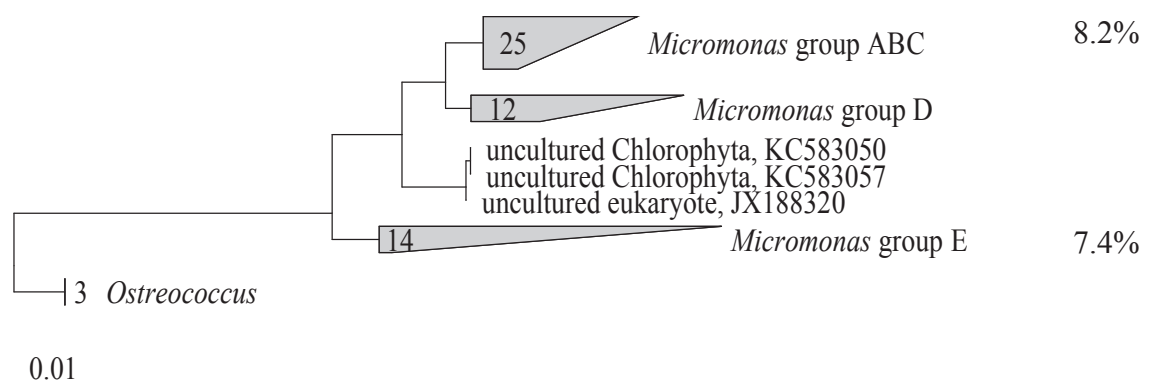

Supplement: Figure S3 — 18S rRNA-based tree reconstruction of the Mamiellales clades. Values in the wedges represent the number of reference sequences, while values behind the clades show the abundance of LOHAFEX sequences in these clades The tree was build using the ARB SILVA ref 119 database [30], calculated using Neighbour Joining and Maximum Likelihood algorithms. The aligned consensus tag sequences were added with parsimony criteria to the trees and percentage of tags falling into the respective clade are given behind the clades. Values in the wedges represent the number of reference sequences. Scale bar represents 1% estimated base substitution. (PDF) [file pone.0113244.s003.pdf]
