# Supplementary material for: Stable Composition of the Nano- and Picoplankton Community during the Ocean Iron Fertilization Experiment LOHAFEX
Source: PLoS One. 2014 Nov 17;9(11):e113244. doi: 10.1371/journal.pone.0113244 (PMC4234645; doi:10.1371/journal.pone.0113244)
Supplement: Table S1 — Summary statistics of pyrosequencing reads. The table also contains values after quality filtering and number of OTUs of the 0.2–5 µm size fraction. (PDF) [file pone.0113244.s004.pdf]

Table S1: Summary of recovered 454-pyrosequencing reads, quality filtering and number of OTUs.

	Sample			
	**-1**	**9**	**18**	**16**
**Total 454-reads**	19138	43623	27735	31504
**Average length (bp)**	326	341	343	344
**Acceptable length***	13536	32538	20967	23934
**Quality filtering:**				
**More than one N, chimeras, incorrect forward primer, and non-target organisms**	1678	3126	2380	2458
**Singletons**	341	826	619	714
**Total filtered reads**	11517	28586	17968	20762
**OTUs (97% identity)**	459	714	599	702
**Abundant OTUs****	12	13	16	12
**Rare OTUs****	447	701	583	690

* reads with a minimum length of 300 bp and a maximum length of 670 bp;

** abundant OTU = number of reads ≥1% of total reads, otherwise it is rare
