# Supplementary material for: Stable Composition of the Nano- and Picoplankton Community during the Ocean Iron Fertilization Experiment LOHAFEX
Source: PLoS One. 2014 Nov 17;9(11):e113244. doi: 10.1371/journal.pone.0113244 (PMC4234645; doi:10.1371/journal.pone.0113244)
Supplement: Table S2 — Total cell numbers. Results of the quantification of all probes at 20 m (2A) and 40 m (2B) depth. Counts for SYN-I-1161 and SYN-II-675 were not determined at day 29 OUT at 20 m and SYN-II-675 were not determined for day 29 OUT at 40 m (n.d.). (PDF) [file pone.0113244.s005.pdf]

Table S2 A: CARD-FISH counts at 20 m depth. Counts for SYN-I-1161 and SYN-II-675 were not determined at day 29 OUT (n.d.).

	Day		EUK516	PRAS04	PRYM02	MICRO1	PHAEO3	PELA02	MAST 1A	MAST 1B	MAST 1C	MAST 3	MAST 4	SYN-I-1161	SYN-II-675
**IN Stations**	**-1**	**Mean**	7.22E+03	3.99E+03	1.27E+03	1.54E+03	1.04E+03	9.08E+02	4.54E+01	4.54E+01	0.00E+00	0.00E+00	0.00E+00	2.27E+02	3.63E+02
		**σ**	3.02E+03	2.57E+02	5.14E+02	6.42E+02	3.21E+02	3.85E+02	6.42E+01	6.42E+01	0.00E+00	0.00E+00	0.00E+00	6.42E+01	0.00E+00
	**5**	**Mean**	3.92E+03	1.55E+03	1.16E+03	9.19E+02	1.35E+03	5.45E+02	2.27E+01	3.40E+01	2.27E+01	0.00E+00	0.00E+00	6.81E+01	1.59E+02
		**σ**	4.81E+01	2.73E+02	6.42E+01	6.26E+02	1.44E+02	4.81E+02	0.00E+00	1.60E+01	0.00E+00	0.00E+00	0.00E+00	0.00E+00	3.21E+01
	**9**	**Mean**	8.06E+02	4.99E+02	6.36E+02	3.52E+02	8.30E+02	3.75E+02	0.00E+00	1.13E+01	1.13E+01	0.00E+00	0.00E+00	4.16E+01	6.81E+01
		**σ**	1.44E+02	2.25E+02	5.46E+02	1.60E+01	5.81E+02	1.60E+01	0.00E+00	1.60E+01	1.60E+01	0.00E+00	0.00E+00	3.34E+01	3.21E+01
	**14**	**Mean**	3.60E+03	7.72E+02	1.02E+03	6.01E+02	1.09E+03	1.70E+02	1.13E+01	5.67E+01	4.54E+01	0.00E+00	1.13E+01	4.65E+01	2.61E+02
		**σ**	6.26E+02	2.57E+02	9.63E+01	3.05E+02	6.42E+01	1.44E+02	1.60E+01	1.60E+01	3.21E+01	0.00E+00	1.60E+01	3.70E+01	1.12E+02
	**18**	**Mean**	3.96E+03	1.03E+03	8.97E+02	7.15E+02	5.79E+02	2.84E+02	0.00E+00	2.27E+01	2.27E+01	0.00E+00	0.00E+00	7.94E+01	1.93E+02
		**σ**	4.33E+02	2.41E+02	3.05E+02	1.60E+01	1.60E+01	3.05E+02	0.00E+00	0.00E+00	0.00E+00	0.00E+00	0.00E+00	1.60E+01	1.60E+01
	**22**	**Mean**	3.99E+03	1.13E+03	1.10E+03	8.40E+02	7.83E+02	8.28E+02	3.40E+01	6.81E+01	2.27E+01	1.13E+01	1.13E+01	1.36E+02	1.25E+02
		**σ**	1.70E+03	3.21E+01	2.41E+02	3.21E+02	1.77E+02	3.05E+02	4.81E+01	3.21E+01	0.00E+00	1.60E+01	1.60E+01	0.00E+00	4.81E+01
	**24**	**Mean**	3.99E+03	7.38E+02	1.12E+03	6.01E+02	5.79E+02	6.13E+02	4.54E+01	1.13E+01	0.00E+00	5.67E+01	1.13E+01	4.54E+01	1.48E+02
		**σ**	8.35E+02	8.02E+01	5.94E+02	2.09E+02	1.60E+01	3.21E+02	3.21E+01	1.60E+01	0.00E+00	4.81E+01	1.60E+01	0.00E+00	8.02E+01
	**33**	**Mean**	3.33E+03	9.31E+02	9.42E+02	8.51E+02	1.26E+03	5.67E+02	4.54E+01	0.00E+00	0.00E+00	0.00E+00	0.00E+00	5.67E+01	1.25E+02
		**σ**	2.07E+03	6.42E+01	2.41E+02	2.09E+02	2.24E+02	3.53E+02	3.21E+01	0.00E+00	0.00E+00	0.00E+00	0.00E+00	1.60E+01	8.02E+01
	**36**	**Mean**	4.72E+03	6.01E+02	1.01E+03	7.04E+02	8.62E+02	4.77E+02	0.00E+00	1.13E+01	0.00E+00	0.00E+00	0.00E+00	5.67E+01	5.67E+01
		**σ**	8.99E+02	4.81E+01	1.12E+02	9.63E+01	1.93E+02	6.42E+01	0.00E+00	1.60E+01	0.00E+00	0.00E+00	0.00E+00	1.60E+01	1.60E+01
**OUT stations**	**-1**	**Mean**	7.22E+03	3.99E+03	1.27E+03	1.54E+03	1.04E+03	9.08E+02	4.54E+01	4.54E+01	0.00E+00	0.00E+00	0.00E+00	2.27E+02	3.63E+02
		**σ**	3.02E+03	2.57E+02	5.14E+02	6.42E+02	3.21E+02	3.85E+02	6.42E+01	6.42E+01	0.00E+00	0.00E+00	0.00E+00	6.42E+01	0.00E+00
	**4**	**Mean**	4.34E+03	1.35E+03	1.15E+03	3.75E+02	7.04E+02	6.36E+02	2.27E+01	2.27E+01	1.13E+01	0.00E+00	2.27E+01	1.13E+02	2.04E+02
		**σ**	4.49E+02	1.77E+02	4.01E+02	4.81E+01	6.42E+01	1.28E+02	3.21E+01	3.21E+01	1.60E+01	0.00E+00	0.00E+00	0.00E+00	3.21E+01
	**16**	**Mean**	3.99E+03	9.42E+02	1.41E+03	4.54E+02	8.85E+02	1.12E+03	2.27E+01	3.40E+01	5.67E+01	1.13E+01	2.27E+01	1.02E+02	1.82E+02
		**σ**	6.74E+02	4.98E+02	6.42E+01	5.14E+02	9.63E+01	1.60E+01	0.00E+00	4.81E+01	8.02E+01	1.60E+01	3.21E+01	1.60E+01	6.42E+01
	**29**	**Mean**	1.11E+03	3.97E+02	3.29E+02	3.63E+02	2.60E+03	2.95E+02	0.00E+00	1.13E+01	4.54E+01	1.13E+01	0.00E+00	n.d.	n.d.
		**σ**	7.06E+02	1.44E+02	4.81E+01	0.00E+00	1.03E+02	1.28E+02	0.00E+00	1.60E+01	3.21E+01	1.60E+01	0.00E+00	n.d.	n.d.
	**38**	**Mean**	8.93E+03	2.19E+03	9.40E+02	1.84E+03	4.38E+02	1.26E+03	0.00E+00	0.00E+00	0.00E+00	1.62E+01	2.27E+01	1.62E+02	1.30E+02
		**σ**	9.86E+02	8.94E+02	9.17E+01	5.46E+02	4.36E+02	4.13E+02	0.00E+00	0.00E+00	0.00E+00	2.29E+01	0.00E+00	0.00E+00	9.17E+01

Table S2 B: CARD-FISH counts at 40 m depth. Counts for SYN-II-675 were not determined at day 29 OUT (n.d.).

	**Day**		**EUK516**	**PRAS04**	**PRYM02**	**MICRO1**	**PHAEO3**	**PELA02**	**MAST 1A**	**MAST 1B**	**MAST 1C**	**MAST 3**	**MAST 4**	**SYN-I-1161**	**SYN-II-675**
**IN Stations**	**-1**	**Mean**	4.39E+03	2.47E+03	1.01E+03	1.02E+03	7.83E+02	5.56E+02	1.13E+01	0.00E+00	1.13E+01	0.00E+00	2.27E+01	4.54E+01	9.08E+01
		**σ**	4.98E+02	7.06E+02	4.81E+01	3.85E+02	8.02E+01	1.12E+02	1.60E+01	0.00E+00	1.60E+01	0.00E+00	3.21E+01	3.21E+01	0.00E+00
	**5**	**Mean**	3.94E+03	1.13E+03	9.65E+02	8.62E+02	9.82E+02	1.11E+03	2.27E+01	2.27E+01	0.00E+00	0.00E+00	0.00E+00	3.40E+01	2.04E+02
		**σ**	5.62E+02	9.63E+01	5.62E+02	4.81E+02	2.66E+02	4.81E+02	0.00E+00	3.21E+01	0.00E+00	0.00E+00	0.00E+00	1.60E+01	0.00E+00
	**9**	**Mean**	3.01E+03	6.81E+02	1.12E+03	5.33E+02	6.47E+02	3.06E+02	1.13E+01	0.00E+00	0.00E+00	0.00E+00	0.00E+00	7.94E+01	1.93E+02
		**σ**	6.26E+02	4.49E+02	4.01E+02	4.33E+02	4.81E+01	2.73E+02	1.60E+01	0.00E+00	0.00E+00	0.00E+00	0.00E+00	1.60E+01	1.60E+01
	**14**	**Mean**	1.38E+03	3.63E+02	4.01E+02	2.84E+02	1.05E+03	2.16E+02	0.00E+00	4.54E+01	2.27E+01	1.13E+01	1.13E+01	5.67E+01	1.82E+02
		**σ**	4.17E+02	3.21E+01	2.57E+02	1.12E+02	4.36E+02	3.05E+02	0.00E+00	3.21E+01	3.21E+01	1.60E+01	1.60E+01	4.81E+01	0.00E+00
	**18**	**Mean**	1.80E+03	1.43E+03	7.38E+02	5.90E+02	5.79E+02	4.88E+02	1.13E+01	2.27E+01	4.54E+01	0.00E+00	0.00E+00	4.54E+01	1.70E+02
		**σ**	9.79E+02	6.42E+01	2.09E+02	1.60E+02	1.60E+01	5.62E+02	1.60E+01	0.00E+00	0.00E+00	0.00E+00	0.00E+00	3.21E+01	4.81E+01
	**22**	**Mean**	5.32E+03	2.53E+03	1.25E+03	1.55E+03	1.01E+03	6.92E+02	3.40E+01	4.54E+01	7.94E+01	0.00E+00	3.40E+01	1.59E+02	3.29E+02
		**σ**	6.58E+02	7.86E+02	9.63E+01	4.98E+02	3.04E+02	1.12E+02	1.60E+01	3.21E+01	4.81E+01	0.00E+00	4.81E+01	0.00E+00	4.81E+01
	**24**	**Mean**	5.52E+03	1.70E+03	1.03E+03	1.28E+03	3.29E+02	6.92E+02	0.00E+00	0.00E+00	1.13E+01	1.13E+01	0.00E+00	1.02E+02	2.38E+02
		**σ**	9.95E+02	6.74E+02	2.09E+02	6.26E+02	1.60E+01	3.37E+02	0.00E+00	0.00E+00	1.60E+01	1.60E+01	0.00E+00	1.60E+01	4.81E+01
	**33**	**Mean**	3.17E+03	5.22E+02	7.83E+02	6.58E+02	6.22E+02	6.92E+02	1.13E+01	1.13E+01	0.00E+00	1.13E+01	0.00E+00	2.27E+01	9.08E+01
		**σ**	8.19E+02	0.00E+00	3.05E+02	9.63E+01	3.62E+02	5.62E+02	1.60E+01	1.60E+01	0.00E+00	1.60E+01	0.00E+00	0.00E+00	3.21E+01
	**36**	**Mean**	2.75E+03	1.13E+03	8.17E+02	8.85E+02	1.82E+03	1.93E+02	0.00E+00	0.00E+00	2.27E+01	1.13E+01	1.13E+01	6.81E+01	1.48E+02
		**σ**	6.42E+02	6.42E+01	9.63E+01	4.81E+02	5.78E+02	2.09E+02	0.00E+00	0.00E+00	3.21E+01	1.60E+01	1.60E+01	3.21E+01	1.60E+01
**OUT Stations**	**-1**	**Mean**	4.39E+03	2.47E+03	1.01E+03	1.02E+03	7.83E+02	5.56E+02	1.13E+01	0.00E+00	1.13E+01	0.00E+00	2.27E+01	4.54E+01	9.08E+01
		**σ**	4.98E+02	7.06E+02	4.81E+01	3.85E+02	8.02E+01	1.12E+02	1.60E+01	0.00E+00	1.60E+01	0.00E+00	3.21E+01	3.21E+01	0.00E+00
	**4**	**Mean**	3.18E+03	9.19E+02	9.65E+02	6.01E+02	7.90E+02	2.61E+02	0.00E+00	2.27E+01	0.00E+00	2.27E+01	0.00E+00	2.27E+01	9.08E+01
		**σ**	1.93E+02	1.44E+02	5.62E+02	4.01E+02	5.91E+00	1.60E+01	0.00E+00	3.21E+01	0.00E+00	3.21E+01	0.00E+00	3.21E+01	9.63E+01
	**16**	**Mean**	4.77E+03	1.41E+03	1.57E+03	5.22E+02	1.24E+03	1.15E+03	3.40E+01	6.81E+01	5.67E+01	2.27E+01	1.13E+01	1.59E+02	2.50E+02
		**σ**	9.63E+01	6.10E+02	4.49E+02	4.17E+02	5.42E+01	3.05E+02	1.60E+01	6.42E+01	4.81E+01	0.00E+00	1.60E+01	6.42E+01	0.00E+00
	**29**	**Mean**	3.05E+03	6.13E+02	1.43E+03	4.20E+02	4.09E+02	6.70E+02	3.40E+01	1.13E+01	4.54E+01	1.13E+01	0.00E+00	1.25E+02	n.d.
		**σ**	9.15E+02	6.42E+01	4.49E+02	2.73E+02	9.63E+01	1.60E+01	4.81E+01	1.60E+01	6.42E+01	1.60E+01	0.00E+00	1.60E+01	n.d.
	**35**	**Mean**	3.20E+03	5.33E+02	8.51E+02	6.36E+02	4.43E+02	5.45E+02	1.13E+01	1.13E+01	0.00E+00	0.00E+00	1.13E+01	1.36E+02	1.59E+02
		**σ**	1.93E+02	2.73E+02	4.01E+02	1.93E+02	1.44E+02	6.42E+01	1.60E+01	1.60E+01	0.00E+00	0.00E+00	1.60E+01	3.21E+01	3.21E+01
	**38**	**Mean**	5.15E+03	4.88E+02	1.16E+03	6.36E+02	1.12E+03	5.90E+02	0.00E+00	3.40E+01	2.27E+01	0.00E+00	0.00E+00	1.48E+02	2.04E+02
		**σ**	5.78E+02	3.05E+02	3.85E+02	1.93E+02	5.27E+02	3.53E+02	0.00E+00	4.81E+01	0.00E+00	0.00E+00	0.00E+00	4.81E+01	3.21E+01
